# Supplementary material for: Design and Economic Assessment of Alternative Evaporation Processes for Poly-Lactic Acid Production
Source: Polymers (Basel). 2022 May 23;14(10):2120. doi: 10.3390/polym14102120 (PMC9143702; doi:10.3390/polym14102120)
Supplement: Supplementary file 1 [file polymers-14-02120-s001.zip › polymers-1719473-supplementary.pdf]

Supplementary Materials

Design and Economic Assessment of Alternative Evaporation Processes for Poly-Lactic Acid Production

Jonghun Lim 1,2,†, Hyungtae Cho 1,†, Kwon-chan Son 3, Yup Yoo 1,2 and Junghwan Kim 1,*

1 Green Materials and Processes R&D Group, Korea Institute of Industrial Technology, 55, Jonga-ro, Ulsan 44413, Korea; ljh94@kitech.re.kr (J.L.); htcho@kitech.re.kr (H.C.); yooyup@kitech.re.kr (Y.Y.)

2 Department of Chemical and Biomolecular Engineering, Yonsei University, 50, Yensei-ro, Seoul 03722, South Korea

3 Advanced Materials Production Dept, KCC Corporation, 19, Wanjusandan 4(sa)-ro, Jeonju 55321, South Ko-rea; kcson@kccword.co.kr

* Correspondence: author: kjh31@kitech.re.kr

† These authors contributed equally to this work.

A. Developed process model of each process

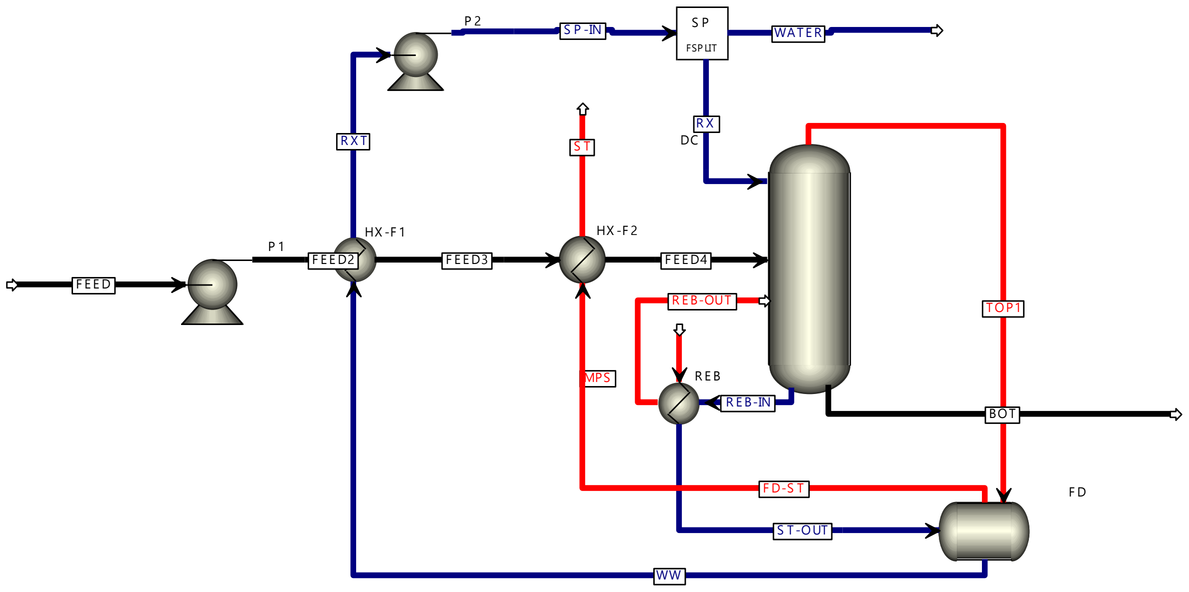

Figure S1. Model of conventional process using SEE

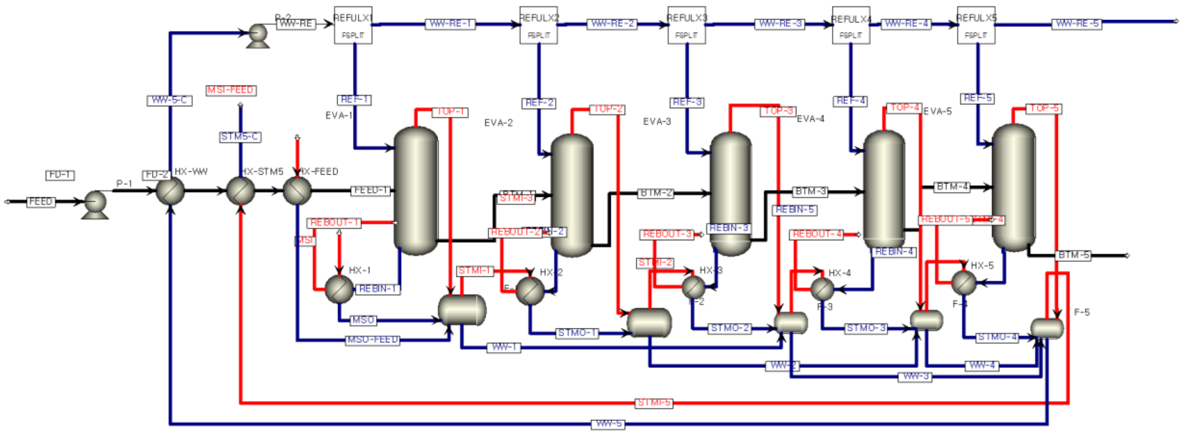

Figure S2. Model of alternative process using MEE

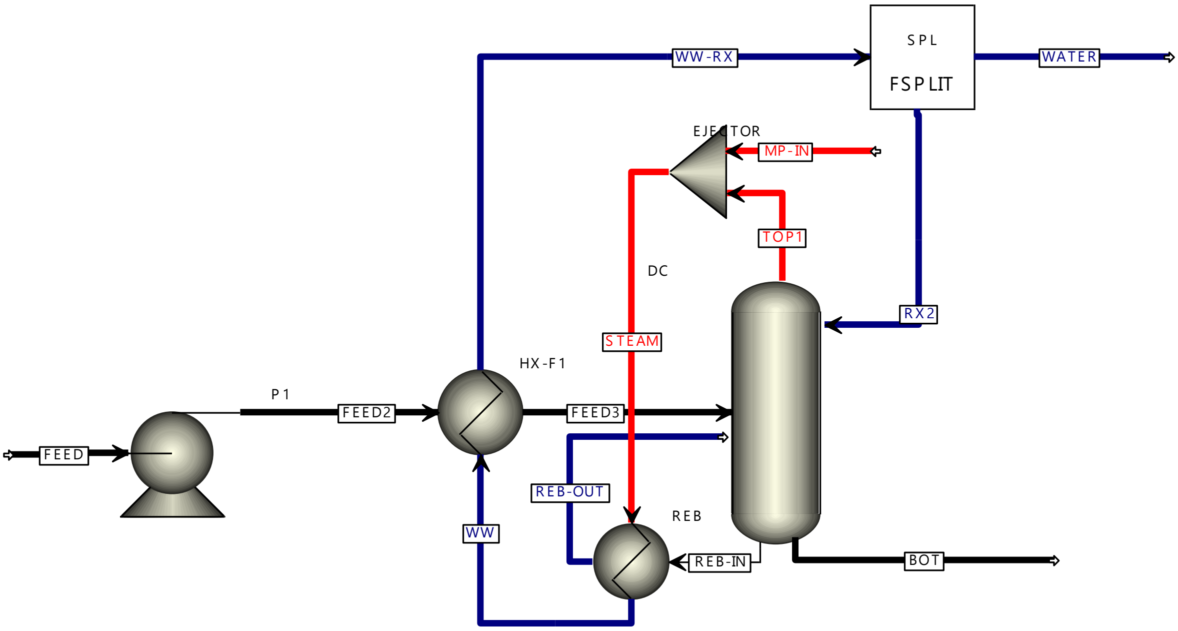

Figure S3. Model of alternative process using TVR

B. Detailed simulation results of each process

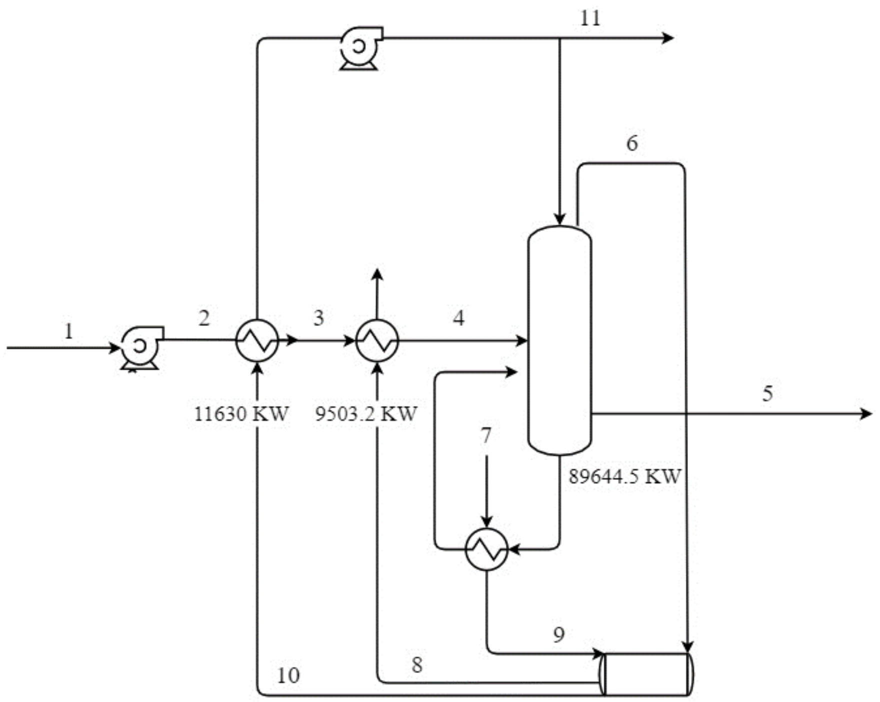

Figure S4. Simplified diagram of the stream number in conventional process using SEE

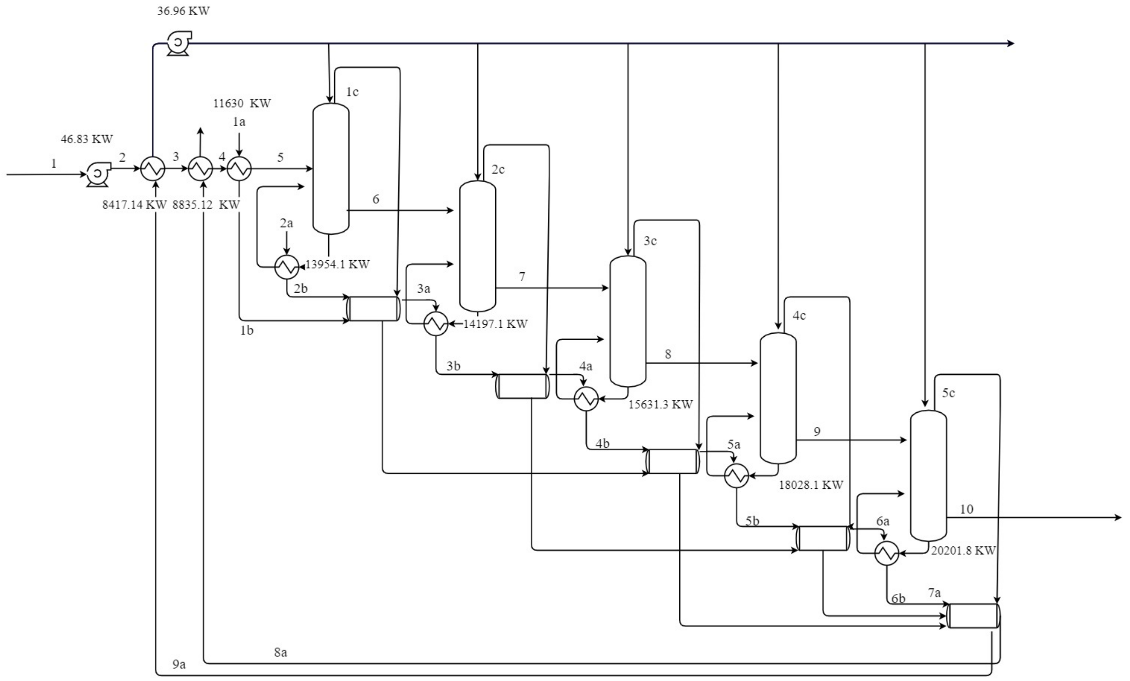

Figure S5. Simplified diagram of the stream number in alternative process using MEE

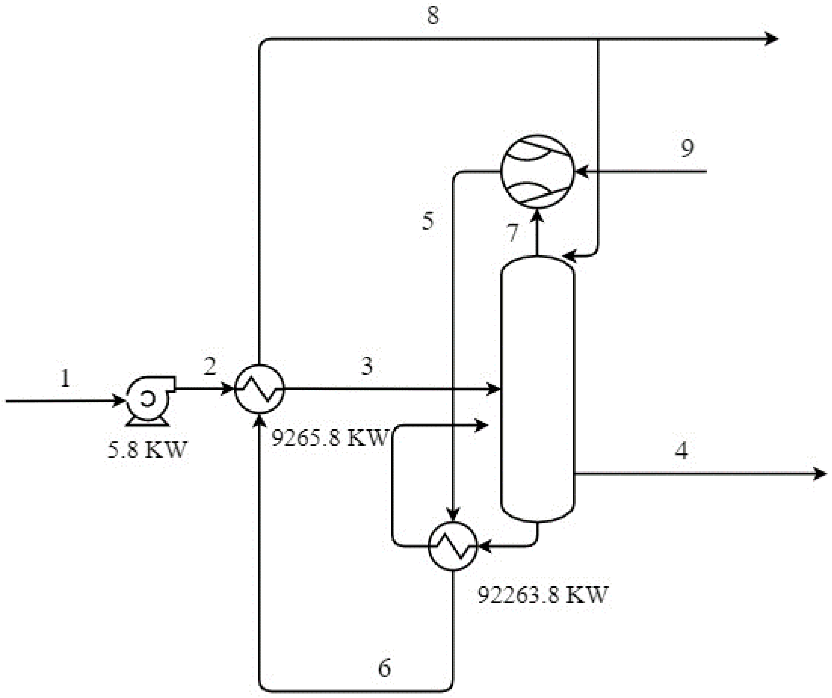

Figure S6. Simplified diagram of the stream number in alternative process using TVR

Table S1. Stream result of the conventional process

Classification	1	2	3	4	5	6	7	8	9	10	11	Units
Phase	Liquid	Liquid	Liquid	Liquid	Liquid	Vapor	Vapor	Vapor	Liquid	Liquid	Liquid	
Temperature	25	25.09	88.37	138.28	159.2	143.3	179.27	143.28	179.27	143.28	77.5	℃
Pressure	0	8	8	8	3.1	3	9	3	9	3	8	$kg/cm^{2}\cdot g$
Mass flows	161,600	161,600	161,600	161,600	21,535	147,565	161,726	161,872	161,726	147,419	139,919	kg/h
Mass fractions												
LA	0.1	0.1	0.1	0.1	0.75	0	0	0	0	0	0	
WATER	0.9	0.9	0.9	0.9	0.25	1	1	1	1	1	1	

Table S2. Stream results of the alternative process using MEE

Classification	1	2	3	4	5	6	7	8	9	10	Units
Phase	Liquid	Liquid	Liquid	Liquid	Liquid	Liquid	Liquid	Liquid	Liquid	Liquid	
Temperature	25.09	70.75	116.7	25	173.53	173.76	166.65	158.51	148.45	134.37	℃
Pressure	8	8	8	0	8	7.6	6.2	4.81	3.35	1.2	$kg/cm^{2}\cdot g$
Mass vapor fraction	0	0	0	0	0	0	0	0	0	0	
Mass liquid fraction	1	1	1	1	1	1	1	1	1	1	
Mass flows	161,600	161,600	161,600	161,600	161,600	138,850	113,650	86,350	55,645	21,356	kg/h
Mass fractions											
LA	0.1	0.1	0.1	0.1	0.1	0.12	0.14	0.19	0.29	0.75	
WATER	0.9	0.9	0.9	0.9	0.9	0.88	0.86	0.81	0.71	0.25	

Table S2. Stream results of the alternative process using MEE (Continued)

Classification	1a	2a	3a	4a	5a	6a	7a	1b	2b	3b	4b	5b	6b	Units
Phase	Vapor	Vapor	Vapor	Vapor	Vapor	Vapor	Vapor						Liquid	
Temperature	179.27	179.27	172.38	165.02	156.47	145.89	121.71	179.27	179.27	172.38	165.01	156.46	145.2	℃
Pressure	9	9	7.5	6.1	4.71	3.3	1.1	9	9	7.5	6.1	4.71	3.3	$kg/cm^2 \cdot g$
Mass vapor fraction	1	1	1	1	1	1	1	0	0	0	0	0	0	
Mass liquid fraction	0	0	0	0	0	0	0	1	1	1	1	1	1	
Mass flows	20,800	25,000	25,156.	27,323	31,065	34,142	45,048	20,800	25,000	25,156	27,323	31,065	34,142	kg/h
Mass fractions														
LA	0	0	0	0	0	0	0	0	0	0	0	0	0	
WATER	1	1	1	1	1	1	1	1	1	1	1	1	1	

Table S2. Stream results of the alternative process using MEE (Continued)

Classification	1c	2c	3c	4c	5c	Units
Phase	Vapor	Vapor	Vapor	Vapor	Vapor	
Temperature	172.53	165.16	156.62	145.59	121.83	℃
Pressure	7.5	6.1	4.71	3.25	1.1	$kg/cm^2 \cdot g$
Mass Vapor Fraction	1	1	1	1	1	
Mass Liquid Fraction	0	0	0	0	0	
Mass Flows	24,300	26,800	28,800	32,300	35,393	kg/h
Mass Fractions						
LA	0	0	0	0	0	
WATER	1	1	1	1	1	

Table S3. Stream results of the alternative process using TVR

Classification	1	2	3	4	5	6	7	8	9	Units
Phase	Liquid	Liquid	Liquid	Liquid			Vapor	Liquid	Vapor	
Temperature	25	25.01	75.53	104.91	123.16	123.16	88.82	80.53	179.27	℃
Pressure	0	1	1	-0.25	1.2	1.2	-0.35	1.2	9	$kg/cm^2{\cdot}g$
Mass Vapor Fraction	0	0	0	0	0.98	0.01	1	0	1	
Mass Liquid Fraction	1	1	1	1	0.02	0.99	0	1	0	
Mass Flows	161600	161600	161600	21546	163770.89	163770.89	147554	163770.89	16216.89	kg/h
Mass Fractions										
WATER	0.9	0.9	0.9	0.25	1	1	1	1	1	
LA	0.1	0.1	0.1	0.75	0.00E+00	0.00E+00	0.00E+00	0.00E+00	0	

C. Detailed description of equivalent annual cost calculation

Table S4. Detailed description of equivalent annual cost calculation

Classification	Remark
**Direct cost onsite**	
Purchased Equipment($)	E
Instruments($)	47% E
Installation($)	18% E
Piping($)	66% E
Electrical($)	11% E
**Direct costs offsite**	
Buildings($)	18% E
Yard Improvements($)	10% E
Service facilites($)	70% E
Land($)	6% E
**Indirect Cost**	
Engineering($)	33% E
construction($)	41% E
Contracter's fee($)	5%(Direct+ Eng + Const)
Contigency($)	10%(Direct + Eng +Const)
**Working Capital**	15% of FCI
**FCI($)**	Direct cost + Indirect cost
**TCI($)**	FCI+ Working capital
**EAC($)**	TCI/AF
